# Telomere length in COPD: Relationships with physical activity, exercise capacity, and acute exacerbations

**DOI:** 10.1371/journal.pone.0223891

**Published:** 2019-10-17

**Authors:** Emily S. Wan, Rebekah L. Goldstein, Vincent S. Fan, Huong Q. Nguyen, Jaime E. Hart, Eric Garshick, Esther H. Orr, Immaculata DeVivo, Marilyn L. Moy

**Affiliations:** 1 VA Boston Healthcare System, Boston, MA, United States of America; 2 Channing Division of Network Medicine, Brigham and Women’s Hospital, Boston, MA, United States of America; 3 Harvard Medical School, Boston, MA, United States of America; 4 VA Puget Sound, Seattle, WA, United States of America; 5 University of Washington, Seattle, WA, United States of America; 6 Department of Research & Evaluation, Kaiser Permanente Southern California, Pasadena, CA, United States of America; 7 Harvard T.H. Chan School of Public Health, Boston, MA, United States of America; 8 Brigham & Women’s Hospital, Boston, MA, United States of America; University of Newcastle, UNITED KINGDOM

## Abstract

**Rationale:**

Shorter leukocyte telomere length (LTL) is associated with reduced health-related quality of life and increased risk for acute exacerbations (AEs) and mortality in chronic obstructive pulmonary disease (COPD). Increased physical activity and exercise capacity are associated with reduced risk for AEs and death. However, the relationships between LTL and physical activity, exercise capacity, and AEs in COPD are unknown.

**Methods:**

Data from 3 COPD cohorts were examined: Cohort 1 (n = 112, physical activity intervention trial), Cohorts 2 and 3 (n = 182 and 294, respectively, separate observational studies). Subjects completed a 6-minute walk test (6MWT) and provided blood for LTL assessment using real-time PCR. Physical activity was measured as average daily step count using an accelerometer or pedometer. Number of self-reported AEs was available for 1) the year prior to enrollment (Cohorts 1 and 3) and 2) prospectively after enrollment (all cohorts). Multivariate models examined associations between LTL and average daily step count, 6MWT distance, and AEs.

**Results:**

A significant association between longer LTL and increased 6MWT distance was observed in the three combined cohorts (β = 3x10^-5^, p = 0.045). No association between LTL and average daily step count was observed. Shorter LTL was associated with an increased number of AEs in the year prior to enrollment (Cohorts 1 and 3 combined, β = -1.93, p = 0.04) and with prospective AEs (Cohort 3, β = -1.3388, p = 0.0003).

**Conclusions:**

Among COPD patients, increased LTL is associated with higher exercise capacity, but not physical activity. Shorter LTL was associated with AEs in a subgroup of cohorts.

## Introduction

Telomeres, which are comprised of repetitive nucleotide sequences and protein complexes, are located at the distal ends of linear eukaryotic chromosomes and protect against chromosomal degradation and the loss of genetic information. Leukocyte telomere length (LTL) is determined by multiple factors. Initially, LTL may be influenced by intrinsic factors such as genetic sequence variation[1, 2] and extrinsic factors such as perinatal and early life exposures[3, 4]. Throughout an individual’s lifetime, aging as well as intrinsic and extrinsic factors progressively shorten LTL–thus, the concept of LTL as an integrative summation of an individual’s “biological age” is appealing[5].

In chronic obstructive pulmonary disease (COPD), telomeres may contribute to disease susceptibility and development[6, 7] in addition to reflecting lifetime exposures such as cigarette smoke[8, 9]. Longitudinal population-based studies have suggested that individuals with shorter telomeres may be more susceptible to the effects of cigarette smoking[6] and multiple cross-sectional studies have confirmed shorter LTL in COPD subjects relative to individuals without airflow limitation[7, 10]. Among subjects with established COPD, decreased LTL is associated with increased airflow limitation, reduced health-related quality of life, accelerated rate of shortening of telomeres, and increased mortality[7, 10–12]. Among a cohort of COPD subjects enriched for a history of acute exacerbations (AEs), shorter LTL was associated with increased risk for future AE[12].

Associations between longer LTL and higher exercise capacity, defined as an individual’s maximal ability to perform work[13], as well as physical activity (PA), defined as any movement that expends energy[13], have been previously reported among older adults without COPD[14, 15]. In COPD, exercise capacity and PA are significant predictors of healthcare utilization and mortality[16, 17]. We and others have shown that persons with COPD with higher levels of PA, a modifiable behavior, have better clinical outcomes, such as better functional status, decreased risk of AEs, and decreased risk of dying, independent of pulmonary function[18–21]. However, the relationship between telomere length, PA and exercise capacity, and clinical events such as AEs in COPD is currently unknown.

In these analyses, we examine data from 3 well-characterized cohorts of COPD patients to explore the biobehavioral relationships between LTL and PA and exercise capacity, and expand upon previous investigations into the relationship between LTL and AEs[12]. We examined the relationship between cross-sectional LTL and (1) directly measured PA, (2) exercise capacity assessed via 6 minute walk test (6MWT), (3) history of AE in the year prior to enrollment, and (4) risk of future AEs.

## Materials and methods

### COPD cohorts and assessments

All participants were ≥40 years old with COPD (≥10 pack-years smoking and forced expiratory volume in the first second (FEV_1_) / forced vital capacity (FVC) ratio<0.70 on spirometry or emphysema on clinical chest CT (for Cohorts 1 and 2)). Exclusion criteria included inability to ambulate, unstable cardiovascular disease, and occurrence of an AE <4 weeks prior to enrollment. Cohort 1 was comprised of 117 subjects recruited from the pulmonary clinics at the VA Boston Healthcare System in 2012–2016 for participation in a 12-week PA intervention trial [22]; data from the baseline visit were used in this analysis. Cohort 2 was comprised of 190 subjects participating in an observational study of PA in COPD subjects and were recruited from VA Boston from 2009–2011[20]. Cohort 3 was an observational study of PA in 294 participants enrolled in the COPD Activity: Serotonin Transporter (SERT), Cytokines and Depression (CASCADE) study from 2010–2016 [23–25]. Spirometry and 6MWT were performed in all cohorts at enrollment in accordance with American Thoracic Society (ATS) guidelines[26, 27]. Similarities and differences in daily step count assessment and criteria for data inclusion for each cohort are shown in Table 1.

**Table 1:** Physical activity assessment details by cohort.

	Cohort 1	Cohort 2	Cohort 3
Accelerometer / Pedometer used	Omron HJ-720 ITC	Omron HJ-720 ITC *and* StepWatch Activity Monitor (SAM)	StepWatch Activity Monitor (SAM)
Valid wear day criteria	≥100 steps and ≥8 hours of wear time	≥200 steps and ≥8 hours of wear time	≥10 hours of wear time
Minimum number of days assessed	≥ 5	≥ 5	≥ 3

Data on AEs were collected (1) retrospectively for the year prior to enrollment and (2) prospectively after enrollment in all cohorts. The methods of assessment, definitions of AEs, and type of AE data (count versus binary) are summarized in Table 2.

**Table 2:** Methods and definitions used in AE assessment by cohort.

	Cohort 1	Cohort 2	Cohort 3
*History of Exacerbations in the Year Prior to Enrollment*			
Method	Self-reported	Self-reported	Self-reported
Period queried	Year prior to enrollment	Year prior to enrollment	Year prior to enrollment
Type of data	Count	Binary (Yes/No)	Count
Question(s) used to assess AE	“Number of exacerbations in the past year”	“Physician diagnosed ‘flare’ of COPD in the past year”	Number of courses of prednisone use in past year
*Future Exacerbations After Enrollment*			
Method	Telephone interview & medical record validation every 3–4 months	Telephone interview & medical record validation every 3–4 months	Telephone interview every 3–4 months
Period of follow-up (years)	1.5 ±0.3	1.2 ±0.3	2.2 ±5.3
Definition / Severity of AE			
**Mild**	Not assessed	Not assessed	Count data available
**Moderate-to-Severe**	Count data available	Count data available	Count data available

Mild exacerbations were defined as ≥2 consecutive days of increased respiratory symptoms. Moderate-to-severe exacerbations were defined as increased symptoms (above) *plus* new antibiotic or systemic steroid use *or* hospitalization.

Study protocols for Cohorts 1 and 2 were approved by VA Boston (IRB Protocols #2328 and #1961, respectively). Cohort 3 was approved by the University of Washington, Seattle (Approval 37332), VA Puget Sound Health Care System (Approval 00240), and the University of Texas Health Science Center at San Antonio/South Texas Veterans Health Care System (Approval HSC20100373H). Written informed consent was obtained from all subjects.

### Telomere length analysis

Blood for LTL analyses was collected at baseline in all cohorts. Genomic DNA was extracted from buffy coat samples. The relative LTL was assessed using a modified, high-throughput real-time quantitative polymerase chain reaction (PCR) assay[28]. All samples were processed and analyzed in a single batch; for specific details regarding LTL data generation, please see the Supplementary Methods (S1 File) [29].

### Statistical analysis

Analyses were performed in the three combined cohorts to assess associations between the entire range of LTL and average daily step count, 6MWT distance, and number of AEs; pooled analyses were adjusted for cohort. Because average daily step count was assessed using two different devices (SAM and Omron monitors), we examined the correlation between LTL and PA 1) separately in each cohort and 2) in pooled groups by device type. Generalized linear models (PROC GLM, SAS version 9.4) assessed for associations between LTL and PA and exercise capacity, adjusting for factors (e.g. age, FEV_1_/FVC, sex, and race) associated with LTL on univariate analyses. Depending upon the distribution of AE data in each cohort, Poisson, zero-inflated Poisson, and negative binomial models adjusting for age, FEV_1_/FVC, sex, non-white race and follow up time (in analyses using prospective data), were constructed to examine the associations between LTL and the number of AEs. In zero-inflated models, FEV_1_% predicted was used as a covariate in the zero model. In Cohort 2, logistic regression was used to analyze the occurrence of AE (assessed as yes/no) in the year prior to study entry. (See Supplementary Methods–S1 File)

## Results

### Cohort characteristics

Participant characteristics by cohort and combined are shown in Table 3. The majority of participants were male (89.5%) and white (89.7%) with extensive lifetime exposure to cigarette smoke. Significant differences in baseline characteristics were observed between the three cohorts. Subjects in Cohort 2 had the highest mean age and cumulative exposure to cigarette smoke (pack-years), while subjects in Cohort 3 had the lowest mean FEV_1_% predicted, 6MWT distance, and LTL. Correlations between baseline PA and 6MWT in each cohorts are shown in Supplementary (S1 Table).

**Table 3:** Participant characteristics by cohort.

	Cohort 1	Cohort 2	Cohort 3	Combined Cohort	p-value
n	117	190	294	601	
Age (years) (n = 600)	68.58 ± 8.37	71.647 ± 8.66	67.62 ± 8.46	69.08 ± 8.68	< .0001
Sex (Male)	115 (98.29)	187 (98.42)	236 (80.27)	538 (89.52)	< .0001
Race (Non-white)	10 (8.55)	12 (6.32)	40 (13.61)	62 (10.32)	0.03
BMI (kg/m2)	28.98 ± 5.54	29.01 ± 6.37	28.13 ± 6.05	28.58 ± 6.06	0.2161
Current Smoker (n = 600)	43 (36.75)	46 (24.34)	80 (27.21)	169 (28.17)	0.06
Pack Years (n = 588)	59.17 ± 40.83	66.09 ± 36.95	56.69 ± 29.51	60.18 ± 34.63	0.0145
FEV_1_ (liters) (n = 594)	1.86 ± 0.61	1.58 ± 0.61	1.27 ± 0.54	1.48 ± 0.62	< .0001
FVC (liters) (n = 594)	3.29 ± 0.79	3.01 ± 0.79	2.97 ± 0.85	2.97 ± 0.85	< .0001
FEV_1_/FVC (n = 594)	0.56 ± 0.11	0.52 ± 0.12	0.45 ± 0.12	0.49 ± 0.13	< .0001
FEV_1_% predicted (n = 593)	61.98 ± 21.28	55.92 ± 20.99	41.8 ± 15.08	50.29 ± 20.26	< .0001
FVC % predicted (n = 593)	80.66 ± 19.46	77.19 ± 19.06	69.25 ± 16.73	74.03 ± 18.65	< .0001
MOS score (n = 599)	3.61 ± 1.12	3.76 ± 1.06	3.72 ± 1.09	3.71 ± 1.09	0.4831
MMRC dyspnea score	1.62 ± 1.14	2.14 ± 1.20	1.91 ± 1.1	1.92 ± 1.15	0.0005
Heart Attack Ever (n = 600)	9 (7.69)	50 (26.46)	46 (15.65)	105 (17.50)	< .0001
Congestive Heart Failure (CHF)	8 (6.84	23 (12.11)	15 (5.10)	46 (7.65)	0.02
Diabetes	29 (24.79)	51 (26.84)	66 (22.45)	146 (24.29)	0.54
Depression	45 (38.46)	52 (27.37)	79 (26.87)	176 (29.28)	0.05
Arthritis	42 (35.90)	97 (51.05)	101 (34.35)	240 (39.93)	0.0007
Use of Oxygen during Rest (n = 600)	7 (5.98)	20 (10.58)	52 (17.69)	79 (13.17)	0.003
6MWT Distance (m)	386 ± 83	369 ± 105	334 ± 113	355 ± 107	< .0001
Mean Baseline Step Count—Omron (n = 292)	3457 ± 2462	2851 ± 2373	---	3084 ± 2421	0.0371
Mean Baseline Step Count—SAM (n = 459)	---	5726 ± 3156	6043 ± 3345	5917 ± 3271	0.3093
Exp ddCt (Telomere Length)	0.54 ± 0.10	0.59 ± 0.10	0.52 ± 0.10	0.54 ± 0.10	< .0001

Data are shown as mean ± SD or n (%). P-values are for Tukey’s test for differences in means by cohort (continuous variables) or Chi-square test (categorical variables). MOS SF-36 = Medical Outcomes Study Short-Form 36 questionnaire. MMRC = modified Medical Research Council. 6MWT = 6-minute walk test. SAM = StepWatch Activity Monitor.

### Telomere length and baseline characteristics

Associations between LTL and clinically relevant continuous and categorical variables are shown in Supplementary (S2 Table). A significant inverse relationship between shorter LTL and higher chronological age at enrollment was observed in the combined cohort. Longer average LTL was significantly associated with non-white race, self-reported history of depression, and higher FEV_1_/FVC ratio in the combined cohort. Current smoking at enrollment was nominally associated with longer average LTL relative to non-current smokers in the combined cohort; however, cumulative pack-years was not significantly associated with LTL in any of the cohorts.

### Telomere length and baseline physical activity

No significant correlations between LTL and average daily step count were identified in the individual cohorts. In combined analyses grouped by PA monitoring device, LTL demonstrated a weak but significant positive correlation with Omron-assessed steps from Cohorts 1 (n = 112) and 2 (n = 180) combined (rho = 0.16, p-value = 0.01). However, this association did not remain significant in multivariate models adjusted for age, sex, non-white race, and FEV_1_/FVC ratio (S3 Table). No correlation between LTL and SAM-assessed average daily step count was observed in either Cohort 2 or 3 individually or in a combined cohort. A post-hoc random-effects meta-analysis, conducted using Omron-assessed daily step count in Cohorts 1 and 2 and SAM-assessed daily step count in Cohort 3, did not demonstrate a significant association between LTL and average daily step count (S4 Table).

### Telomere length and baseline exercise capacity

In the combined cohort, a positive correlation (rho = 0.15, p = 0.0002) between longer LTL and greater 6MWT distance was observed, which remained significant in multivariate models adjusting for age, sex, race, and FEV_1_/FVC ratio (Table 4).

**Table 4:** Multivariate model of baseline leukocyte telomere length and 6-minute walk test distance (meters) in a combined cohort (Cohorts 1, 2, and 3).

Continuous measures	β	95% CI	p-value
6MWT Distance (m)	0.00003	0.00, 0.00	0.045
Age	-0.0019	-0.003, -0.001	0.0002
FEV_1_/FVC	0.1167	0.05, 0.18	0.001
Categorical measures	Least Square Means	95% CI	p-value
Race			0.002
White	0.5346	0.52, 0.55	
Non-white	0.5800	0.55, 0.61	
Sex			0.847
Male	0.5560	0.54, 0.57	
Female	0.5586	0.53, 0.59	

6MWT = 6-minute walk test. Generalized linear models were constructed with leukocyte telomere length as the independent variable and 6MWT as the independent variable, with adjustment for age, FEV_1_/FVC ratio, non-white race, and sex.

### Telomere length and acute exacerbations in the year prior to enrollment

Since historical AE frequency was assessed differently in the 3 cohorts, separate models were used in each cohort. Shorter LTL at baseline was significantly associated with a greater number of AEs in the year prior to enrollment in Cohort 1 (Table 5). Using a similar model, there was a similar direction of effect with a trend towards significance in Cohort 3 (p = 0.1). A significant association between shorter LTL and greater number of AEs in the year prior to enrollment was observed in a combined analysis of both Cohorts 1 and 3. No significant association was observed between LTL and binary history of AE (yes/no) in the year prior to enrollment in Cohort 2. When we examined dichotomized history of AEs (yes/no) in all 3 cohorts combined, no significant association with LTL was observed.

**Table 5:** Association between baseline leukocyte telomere length and number of acute exacerbations in the year prior to enrollment.

	Coeff	95% CI	p-value
Cohort 1	-8.31	-15.41, -1.20	0.02
Cohort 3	-1.58	-3.45, 0.28	0.1
Combined (Cohorts 1 & 3)*	-1.93	-3.72, -0.13	0.04

Coeff = Regression coefficient. Zero-inflated Poisson models were constructed for each analysis with telomere length as the independent variable and the number of acute exacerbations in the year prior to enrollment as the dependent variable. All analyses included adjustment for age, FEV_1_/FVC ratio, non-white race, and sex; FEV _1_% predicted was included as a predictor in the zero model. Each row of the table represents the results of a separate model.

* Model additionally adjusted for cohort as a categorical variable.

### Telomere length and future acute exacerbation risk

**Fig 1:**
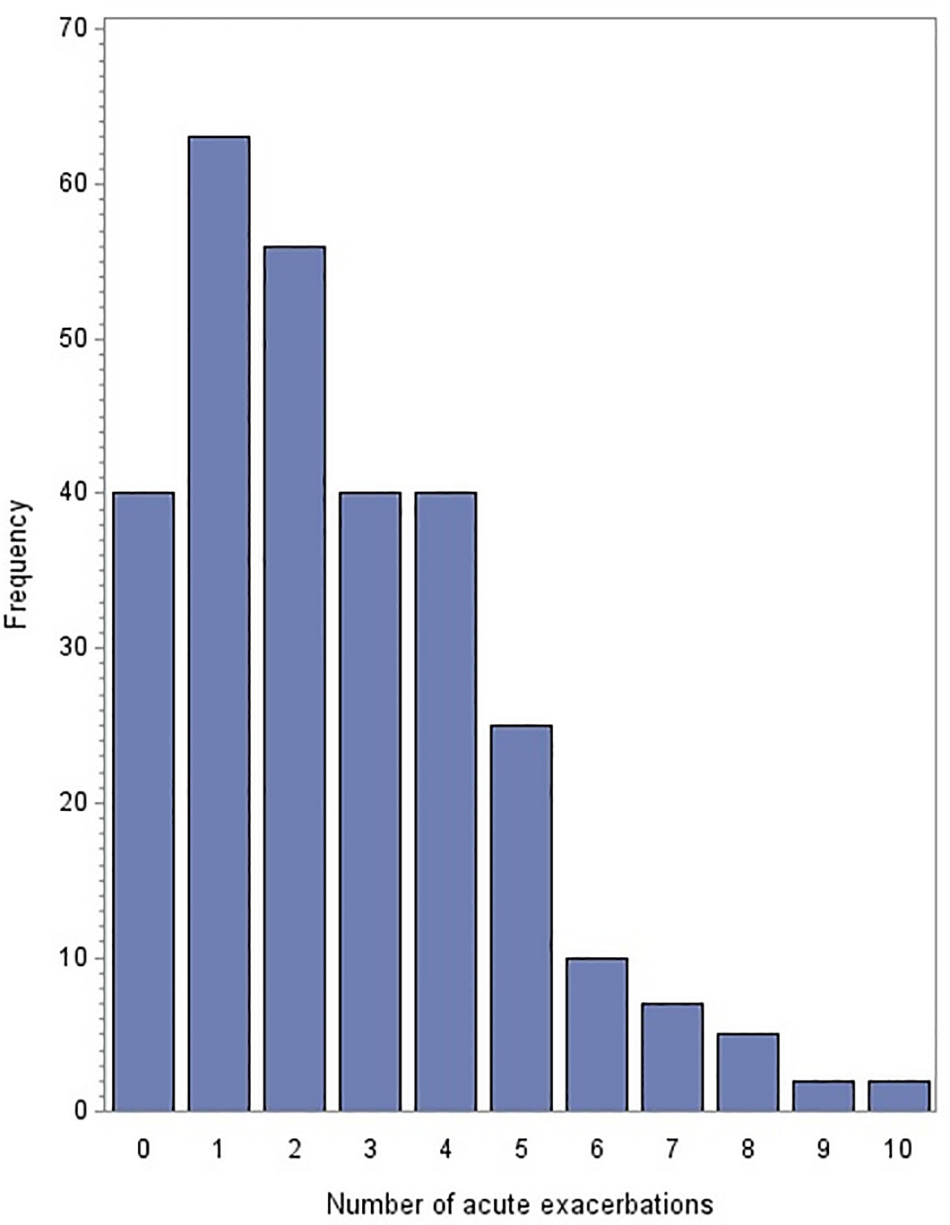
Distribution of all prospective acute exacerbations (mild-moderate-severe) in Cohort 3.

The average duration of follow-up for all cohorts combined was 1.8 ±3.8 years with significant differences by cohort (Table 2); duration of follow-up was used as an offset in all models. The distribution of all moderate-to-severe AEs in the combined cohort is shown in Supplementary (S1 Fig); based on this distribution, a negative binomial model was constructed with the number of AEs as the dependent variable and LTL as the predictor, with covariate age, FEV1% predicted, and cohort–no significant associations were observed (S5 Table). However, given the evidence of significant heterogeneity between cohorts, we examined for associations within each cohort separately. The distribution of moderate-to-severe AEs in each individual cohort is shown in Supplementary (S2 Fig). Separate negative binomial regressions in Cohorts 1, 2, and 3 did not demonstrate any associations between moderate-to-severe AEs and LTL. Because additional data on *mild* AEs was available in Cohort 3, we examined the distribution of all prospective AEs (mild-moderate-severe; Fig 1) and performed a subgroup analysis using a multivariate Poisson regression and observed a significant association between LTL and total AEs (Table 6).

**Table 6:** Multivariate Poisson regression of baseline leukocyte telomere length and number of prospective acute exacerbations (mild-moderate-severe inclusive) after study enrollment in Cohort 3.

	Coeff	95% CI	p-value
Continuous variables			
Leukocyte telomere length	-1.34	-2.07, -0.61	0.0003
FEV_1_% predicted (post bronchodilator)	-0.02	-0.02, -0.01	<0.001
Age	-0.01	-0.02, -0.003	0.0098
Categorical variables			
Race (reference = white)	-0.19	-0.44, 0.07	0.1455
Sex (reference = male)	0.52	0.36, 0.68	<0.0001
Current smoking (reference = no)	0.14	-0.02, 0.31	0.0923

Poisson regression was constructed using leukocyte telomere length as the independent variable and number of all acute exacerbations (mild-moderate-severe) as the dependent variable, with covariates listed above. Follow-up time in years was included as the offset. Coeff = regression coefficient

## Discussion

Accelerated cellular senescence has been postulated to contribute to the pathogenesis of COPD[30–32]. Reduced LTL, often considered a biomarker for biological aging, has been associated with exposure to oxidative and inflammatory damage as well as poor health outcomes and increased mortality among COPD patients[12, 33–35]. Healthy lifestyle factors, such as engagement in PA, are associated with reduced levels of inflammatory biomarkers and risk for AEs in COPD patients[18, 20, 21, 36]. Our study demonstrates that LTL is associated with exercise capacity, and both past and future AEs among subgroups of participants with COPD from 3 independent studies based in the United States.

A key finding was a significant association between baseline LTL and exercise capacity, assessed as the 6MWT distance, after adjusting for age and severity of airflow obstruction. Previous studies have demonstrated positive associations between LTL and cardiopulmonary fitness and maximal aerobic capacity (VO_2_max) among healthy adults who regularly engaged in exercise[14, 37]. Studies of LTL among COPD populations using functional exercise capacity assessments (such as 6MWT) have not been reported previously. Taken together, previous studies and our current results suggest that although LTL may be a possible molecular mechanism through which exercise capacity and mortality may be linked. The impact of sustained engagement in endurance exercise on LTL is unknown. In light of the fact that exercise capacity is both modifiable and the target of training programs such as pulmonary rehabilitation, future longitudinal studies should examine whether sustained aerobic training impacts long-term change in LTL in COPD patients.

Our analyses also support significant associations between shorter LTL and higher number of past and future AEs among several cohorts within our study. An inverse relationship between LTL and history of AE frequency in the year prior to enrollment was observed in the cohorts where quantitative retrospective data was available. The lack of association in the combined (Cohorts 1,2, & 3) analysis of dichotomized AEs (yes/no) in the year prior to enrollment is likely due in part to the reduced power of the binary phenotype. Interestingly, shorter LTL at baseline was also predictive of future AEs in Cohort 3. These results are consistent with those from a study designed to examine the efficacy of chronic macrolide therapy on AE’s in COPD; a higher rate of prospectively assessed moderate-to-severe AE were associated with decreased LTL in the placebo arm of that trial [12]. No association between LTL and future AEs was observed in Cohorts 1 and 2 in our analyses. This may be due to 1) the longer average duration of follow up in Cohort 3, and/or 2) the broader definition of AE used in Cohort 3 (mild, moderate, and severe) relative to Cohorts 1 & 2 (moderate-to-severe AE defined as increased symptoms requiring systemic steroid and/or antibiotic use). It is not possible to infer from our cross-sectional data whether AEs reduce LTL or whether LTL simply serves as a marker of increased susceptibility towards AEs.

Despite a robust association between exercise capacity and LTL, we did not observe an association between LTL and objectively assessed PA. Factors which may have contributed to the lack of association between LTL and PA in our study include heterogeneity between cohorts with respect to study participants and PA assessment devices. Although our study was sufficiently powered to detect modest correlations (>80% power to detect a correlation of 0.15–0.20 at an alpha level of 0.05), the existence of a threshold intensity or duration of PA required to impact LTL[38] may have precluded detection of an association in our studies. Alternatively, factors other than LTL, such as lung disease severity or unmeasured environmental, behavioral, and social factors, may play a larger role determining PA relative to biological or intrinsic physiological factors (e.g. cardiac output, muscle fiber oxidative capacity) among COPD patients.

In conclusion, we have demonstrated significant associations between LTL and exercise capacity and AEs. The strengths of our analyses include a biobehavioral approach to understanding PA and exercise, large number of well-characterized COPD subjects with rigorously assessed PA data, and standardized assessments of functional exercise capacity, spirometry, and comorbidities. Limitations include a predominance of males and limited representation of individuals of non-white ancestry, both of which may limit the generalizability of our findings. Cohort heterogeneity, such as differences in airflow severity, comorbidities, and baseline PA levels, as well as the use of different PA data collection devices and AE ascertainment criteria may have limited the ability to detect significant associations. However, when applicable, we have applied established statistical methods to account for these cohort differences and believe our findings support the need for additional investigations to examine the effects of PA promotion on LTL and the utility of telomeres in the prognostication of COPD-related phenotypes. Future work is needed to build on our preliminary results to understand the biobehavioral basis of the relationships between physical activity, exercise capacity, and telomere length.

## Supporting information

S1 File Supplementary Methods. (DOCX)

S1 Table Spearman correlation between physical activity (average daily step count) and exercise capacity (6 minute walk test) by cohort. (DOCX)

S2 Table Associations between telomere length and baseline characteristics. (XLSX)

S3 Table Multivariable model of baseline leukocyte telomere length and Omron-assessed average daily step counts (combined Cohorts 1 and 2, n = 291). (DOCX)

S4 Table Random-effects meta-analysis of physical activity and leukocyte telomere length in Cohorts 1, 2, and 3. (DOCX)

S5 Table Negative binomial model of the association between leukocyte telomere length and number of prospective moderate-to-severe acute exacerbations in the combined cohort (Cohorts 1,2,&3). (DOCX)

S1 Fig Distribution of prospective moderate-to-severe acute exacerbations in the combined cohort (Cohorts 1, 2, & 3). (PNG)

S2 Fig Distribution of number of prospective moderate-to-severe AEs after study enrollment in (a) Cohort 1, (b) Cohort 2, and (c) Cohort 3.
(DOCX)
