# Supplementary material for: Telomere length in COPD: Relationships with physical activity, exercise capacity, and acute exacerbations
Source: PLoS One. 2019 Oct 17;14(10):e0223891. doi: 10.1371/journal.pone.0223891 (PMC6797105; doi:10.1371/journal.pone.0223891)
Supplement: S1 File — (DOCX) [file pone.0223891.s001.docx]

**Supplementary Methods**

**Telomere Length Assay**

Five nanograms of genomic DNA were dried down in each 384-well plate and resuspended in 10µL of either the telomere or 36B4 PCR reaction mixture and stored at 4^°^C up to 6 hours. The telomere reaction mixture consisted of 1x Thermo Fisher PowerUP SYBR Master Mix, 2.0mM of DTT, 270nM of Tel-1b primer, and 900nM of Tel-2b primer. The reaction proceeded for 1 cycle hold at 50°C for 2 minutes and at 95°C for 2 minutes, followed by 35 cycles at 95°C for 15 seconds, and 54°C for 2 minutes. The 36B4 reaction consisted of 1x Thermo Fisher PowerUP SYBR Master Mix, 300nM of 36B4U primer, and 500nM of 36B4D primer. The 36B4 reaction proceeded for 1 cycle hold at 50°C for 2 minutes and at 95°C for 2 minutes, followed by 40 cycles at 95°C for 15 seconds, and 58°C for 1 minute and 10 seconds. All samples for both the telomere and single-copy gene (36B4) reactions were performed in triplicate on different plates. Each 384-well plate also contained a 6-point standard curve from 0.625 ng to 20 ng using pooled buffy-coat derived genomic DNA.

The standard curve assessed and compensated for inter-plate variations in PCR efficiency. The slopes of the standard curve for both the telomere and 36B4 reactions were -3.33±0.33 and the linear correlation coefficient (R^2^) values for both reactions were over 0.99. The T/S ratio (-dCt) for each sample was calculated by subtracting the average 36B4 Ct value from the average telomere Ct value. The relative T/S ratio (-ddCt) was determined by subtracting the T/S ratio value of the 5 ng standard curve point from the T/S ratio of each unknown sample. Quality control samples were interspersed throughout the test samples in order to assess inter-plate and intra-plate variability of threshold cycle (Ct) values. A combined inter- and intra-assay coefficient of variation (CV) calculated from the relative T/S ratio (-ddCt) of quality control samples is 8.5%.

**Statistical Models for Acute Exacerbation Analyses**

For analyses of number of AEs reported in the year prior to study entry, the following zero-inflated Poisson model was used to analyse data in Cohorts 1 and 3 separately:

[number of AEs in year prior to enrollment] ~ [telomere length] + [age in years] + [FEV_1_/FVC ratio] + [non-white race] + [gender]

FEV_1_ % predicted was modelled in the zero component. Cohorts 1 and 3 were analysed in a combined cohort using the above model with additional adjustment for cohort (as a factor covariate).

In Cohort 2, where binary (yes/no) history of AEs was available, the following logistic regression was used:

[number of AEs in year prior to enrollment] ~ [telomere length] + [age in years] + [FEV_1_/FVC ratio] + [non-white race] + [gender]

For analysis of the number prospective moderate-to-severe AEs in the combined cohort (Cohorts 1, 2, and 3), the following negative binomial model was used:

[number of AEs after enrollment] ~ [telomere length] + [age in years] + [FEV_1_ % predicted] + [cohort]

Log follow-up time in years was included as an offset.

For analysis number of *all* AEs (mild-moderate-severe) that occurred after study entry in Cohort 3, the following Poisson model was used:

[number of AEs after enrollment] ~ [telomere length] + [age in years] + [FEV_1_/FVC ratio] + [non-white race] + [gender] + [current smoking status]

In Cohorts 1 and 2, only data on moderate-to-severe AEs were available. Separate zero-inflated Poisson models were used in each cohort with the Poisson component identical to the above and additional covariate of FEV_1_ % predicted modelled in the zero component. The log of follow-up time (in years) was included as an offset in all models.
