# Supplementary material for: Telomere length in COPD: Relationships with physical activity, exercise capacity, and acute exacerbations
Source: PLoS One. 2019 Oct 17;14(10):e0223891. doi: 10.1371/journal.pone.0223891 (PMC6797105; doi:10.1371/journal.pone.0223891)
Supplement: S1 Table — (DOCX) [file pone.0223891.s002.docx]

**Supplementary Table S1** – Spearman correlation between physical activity (average daily step count) and exercise capacity (6 minute walk test) by cohort.

|  | Device Type | | | |
| --- | --- | --- | --- | --- |
|  | Stepwatch Activity Monitor | | Omron HJ-720 ITC | |
|  | ρ | p-value | ρ | p-value |
| Cohort 1 | NA | -- | 0.53 | <0.0001 |
| Cohort 2 | 0.63 | <0.0001 | 0.72 | <0.0001 |
| Cohort 3 | 0.55 | <0.0001 | NA | -- |
