# Supplementary material for: Telomere length in COPD: Relationships with physical activity, exercise capacity, and acute exacerbations
Source: PLoS One. 2019 Oct 17;14(10):e0223891. doi: 10.1371/journal.pone.0223891 (PMC6797105; doi:10.1371/journal.pone.0223891)
Supplement: S3 Table — (DOCX) [file pone.0223891.s004.docx]

**Supplementary Table S3** - Multivariable model of baseline leukocyte telomere length and Omron-assessed average daily step counts (combined Cohorts 1 and 2, n=291)

| Continuous measures | β | 95% CI | p-value |
| --- | --- | --- | --- |
| Age | -0.003 | -0.004, -0.001 | 0.0001 |
| FEV_1_/FVC | 0.0143 | -0.077, 0.106 | 0.76 |
| Baseline Daily Step Count (Omron) | 1.23e^-6^ | -3.57e^-6^, 6.03e^-6^ | 0.61 |
| Categorical measures | LS Means (Average LTL) | 95% CI | p-value |
| Race |  |  | 0.16 |
| White | 0.5485 | 0.51, 0.59 |  |
| Non-white | 0.5789 | 0.53, 0.63 |  |
| Sex |  |  | 0.61 |
| Male | 0.5747 | 0.55, 0.60 |  |
| Female | 0.5527 | 0.47, 0.64 |  |
