# Supplementary material for: Telomere length in COPD: Relationships with physical activity, exercise capacity, and acute exacerbations
Source: PLoS One. 2019 Oct 17;14(10):e0223891. doi: 10.1371/journal.pone.0223891 (PMC6797105; doi:10.1371/journal.pone.0223891)
Supplement: S4 Table — (DOCX) [file pone.0223891.s005.docx]

**Supplementary Table S4** - Random-effects meta-analysis of physical activity and leukocyte telomere length in Cohorts 1, 2, and 3.

|  | Estimate | 95% CI | p-value |
| --- | --- | --- | --- |
| Continuous measures |  |  |  |
| Age | -0.002 | -0.003, -0.001 | <0.0001 |
| FEV_1_/FVC | 0.073 | -0.009, 0.047 | 0.04 |
| Baseline Daily Step Count* | 1.84e^-6^ | -4.75e^-6^, 1.038e^-6^ | 0.22 |
| Categorical measures |  |  |  |
| Race (reference: White) | 0.043 | 0.014, 0.071 | 0.003 |
| Sex (reference: Male) | 0.019 | -0.009, 0.047 | 0.18 |

Analysis was adjusted for cohort.

*Physical activity was assessed using Omron-assessed average daily step count in Cohorts 1 & 2 and SAM-assessed average daily step count in Cohort 3.
