# Supplementary material for: Telomere length in COPD: Relationships with physical activity, exercise capacity, and acute exacerbations
Source: PLoS One. 2019 Oct 17;14(10):e0223891. doi: 10.1371/journal.pone.0223891 (PMC6797105; doi:10.1371/journal.pone.0223891)
Supplement: S5 Table — (DOCX) [file pone.0223891.s006.docx]

**Supplementary Table S5** – Negative binomial model of the association between leukocyte telomere length and prospective moderate-to-severe acute exacerbations in the combined cohort (Cohorts 1,2,&3).

|  | Estimate | 95% CI | p-value |
| --- | --- | --- | --- |
| Continuous measures |  |  |  |
| Telomere length | 0.08 | -1.11, 1.27 | 0.90 |
| Age | -0.01 | -0.02, 0.01 | 0.31 |
| FEV1 % predicted | -0.02 | -0.02, -0.01 | <0.0001 |
| Categorical measures |  |  |  |
| Cohort 1 (reference: Cohort 2) | -1.00 | -1.29, -0.71 | <0.0001 |
| Cohort 3 (reference: Cohort 2) | -0.72 | -1.09, -0.35 | 0.0001 |

Duration of follow-up was include as an offset.
