# Supplementary figures and images for: Telomere length in COPD: Relationships with physical activity, exercise capacity, and acute exacerbations
Source: PLoS One. 2019 Oct 17;14(10):e0223891. doi: 10.1371/journal.pone.0223891 (PMC6797105; doi:10.1371/journal.pone.0223891)

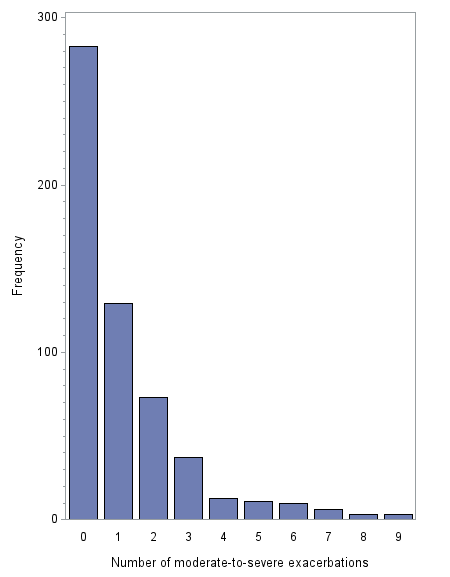

Supplement: S1 Fig — (PNG) [file pone.0223891.s007.png]

(a)
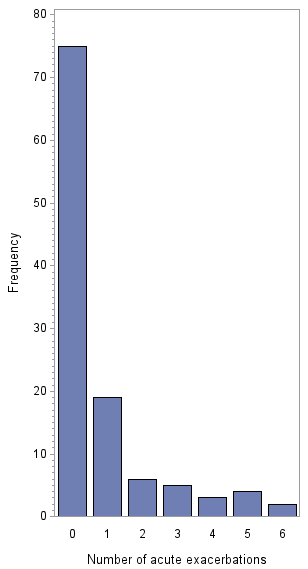
 (b)
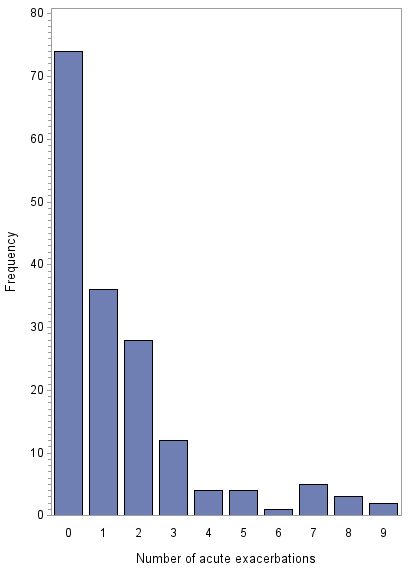
 (c)
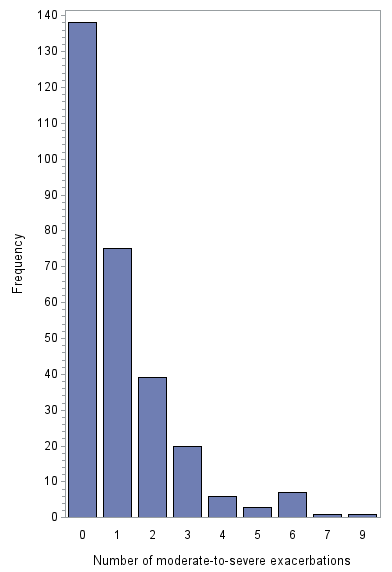

Supplement: S2 Fig — Distribution of number of prospective moderate-to-severe AEs after study enrollment in (a) Cohort 1, (b) Cohort 2, and (c) Cohort 3. (DOCX) [file pone.0223891.s008.docx]
